# Transgenic expression of *Map3k4* rescues *T*-associated sex reversal (*Tas*) in mice

**DOI:** 10.1093/hmg/ddu020

**Published:** 2014-01-22

**Authors:** Nick Warr, Pam Siggers, Gwenn-Aël Carré, Debora Bogani, Rachel Brixey, Mika Akiyoshi, Makoto Tachibana, Lydia Teboul, Sara Wells, Jeremy Sanderson, Andy Greenfield

**Affiliations:** 1 Mammalian Genetics Unit and; 2 The Mary Lyon Centre, Medical Research Council, Harwell, Oxfordshire OX11 0RD, UK,; 3 Experimental Research Center for Infectious Diseases, Institute for Virus Research and; 4 Graduate School of Biostudies, Kyoto University, 53 Shogoin, Kawara-cho, Sakyo-ku, Kyoto 606-8507, Japan

## Abstract

Disorders of sex development in the human population range in severity from mild genital defects to gonadal sex reversal. XY female development has been associated with heterozygous mutations in several genes, including *SOX9*, *WT1* and *MAP3K1*. In contrast, XY sex reversal in mice usually requires complete absence of testis-determining gene products. One exception to this involves *T*-associated sex reversal (*Tas*), a phenomenon characterized by the formation of ovotestes or ovaries in XY mice hemizygous for the hairpin-tail (*T^hp^*) or *T*-Orleans (*T^Orl^*) deletions on proximal mouse chromosome 17. We recently reported that mice heterozygous for a null allele of *Map3k4,* which resides in the *T^hp^* deletion, exhibit XY ovotestis development and occasional gonadal sex reversal on the sensitized C57BL/6J-Y^AKR^ (B6-Y^AKR^) genetic background, reminiscent of the *Tas* phenotype. However, these experiments did not exclude the possibility that loss of other loci in the *T^hp^* deletion, or other effects of the deletion itself, might contribute to *Tas*. Here, we show that disruption to *Sry* expression underlies XY gonadal defects in B6-Y^AKR^ embryos harbouring the *T^hp^* deletion and that a functional *Map3k4* bacterial artificial chromosome rescues these abnormalities by re-establishing a normal *Sry* expression profile. These data demonstrate that *Map3k4* haploinsufficiency is the cause of *T-*associated sex reversal and that levels of this signalling molecule are a major determinant of the expression profile of *Sry*.

## INTRODUCTION

In humans, 46,XY gonadal dysgenesis (46,XY GD) is characterized by abnormal testis determination and is an example of a wider class of abnormalities known as disorders of sex development (DSD). In cases of pure or complete gonadal dysgenesis (CGD), the testes are absent and bilateral streak gonads are observed. Molecular genetic studies of individuals with 46,XY GD and CGD have played a critical role in the identification of human testis-determining genes such as *SRY* (1), *SOX9* (2,3), *WT1* (4) and *SF1* (5). In all autosomal cases, mutation of one copy of the gene can be sufficient to disrupt testis development in some individuals.

XY gonadal sex reversal in the mouse has been described in cases of homozygous deletion of a number of testis-determining genes, including *Sox9* (6–8), *Fgf9* (9), *Fgfr2* (10,11), *Cbx2 (M33)* (12,13) and *Wt1(+KTS)* (14). The C57BL/6J (B6) background is sensitized to disruptions to testis determination due to the relatively delayed expression of testis-determining genes and higher levels of expression of ovary-determining genes and, in most sex-reversing mouse mutants examined, B6 increases the amount of ovarian tissue that forms in mutant XY embryos (15–17). This sensitivity increases still further if an AKR/J-derived Y chromosome (Y^AKR^) is present, resulting in sex reversal even in cases of haploinsufficiency of testis-determining genes, such as *Gata4* and *Fog2* (18). These latter studies suggest that no inherent species differences exist in sensitivity to disruption to testis determination by altered gene dosage; rather, differing sensitivity to gene dosage in both humans and mice is dependent on genetic background.

A number of additional cases of XY sex reversal in the mouse depend on the presence of a *Mus domesticus* Y chromosome and the C57BL/6J genetic background: so called B6.Y^DOM^ sex reversal (19,20). One variant of this phenomenon, known as *T*-associated sex reversal (*Tas*), requires a *domesticus* Y chromosome from the AKR/J strain (Y^AKR^) in combination with a dominant brachyury (*T*) mutation, either hairpin-tail (*T^hp^*) or *T*-Orleans (*T^Orl^*), on the B6 background (21–23). The latter *T* mutations are both caused by overlapping deletions of proximal mouse chromosome 17. Analysis of embryonic gonads revealed that XY *T^hp^*^/+^ individuals develop ovotestes or ovaries, the extent of sex reversal depending on the number of backcrosses performed to B6 (21,23). Ovotestes tend to resolve as development proceeds and form hypoplastic testes, permitting the identification of occasional fertile males (23). Studies of XY gonad development in sex-reversed *T^Orl^* carriers also reveal delayed and significantly reduced levels of *Sry* expression (24). Thus, it has been suggested that haploinsufficiency of a gene (or genes) located in a region common to the *T^hp^* and *T^Orl^* deletions, and required for normal timing and levels of *Sry* expression, is the cause of *Tas*. However, alternative explanations have been proposed, including the possibility that delayed growth in embryos simply harbouring chromosomal deletions such as *T^hp^* and *T^Orl^* might disrupt testis determination, irrespective of the specific gene content of the deletions (25).

We have previously described XY embryonic gonadal sex reversal in embryos lacking *Map3k4,* which encodes a signalling molecule in the mitogen-activated protein kinase (MAPK) pathway (26). Embryos lacking MAP3K4 exhibit reduced *Sry* expression at 11.5 days *post coitum* (dpc) and this results in failure to up-regulate expression of genes functioning in the genetic pathway of testis development, including the key gene, *Sox9*. These data suggested a role for MAPK signalling in the control of *Sry* expression, and thus testis determination. Moreover, the report of mutations in the human *MAP3K1* gene associated with 46,XY DSD and CGD suggests that MAPK signalling is an important component of human testis determination too (27). More recently, we have described data suggesting that disruption to a number of elements of a GADD45γ/MAP3K4/p38 MAPK signalling pathway in somatic cells of the newly formed mouse gonad can result in a delay in *Sry* expression that results in XY gonadal sex reversal (28).

*Map3k4* maps close to *T* on the proximal region of mouse chromosome 17 and a genetic test indicated that it resides in the region deleted in *T^hp^* (26). Thus, *Map3k4* is an outstanding candidate for a gene, haploinsufficiency for which causes *T*-associated sex reversal. Mice heterozygous for a targeted allele of *Map3k4* (*Map3k4^tm1Flv^*) on B6-Y^AKR^ display testicular hypoplasia and occasional XY female development, similar to that described in *Tas* mice. Moreover, heterozygous XY embryos frequently developed ovotestes by 14.5 dpc, consistent with a delay and/or reduction in expression of *Sry* and *Sox9*, although gene expression in the developing gonads at the sex-determining stage (11.5 dpc) was not tested in affected individuals (26). Thus, loss of a single copy of *Map3k4* would be predicted to contribute significantly to the XY sex reversal and ovotestis development observed in *T^hp^*^/+^ C57BL/6J-Y^AKR^ mice. However, whether *Map3k4* is the only gene in the *T^hp^* deletion that predisposes to XY sex reversal when haploinsufficient is unclear from these experiments. That question can only be addressed by determining whether additional functional copies of *Map3k4* can rescue the sex-reversal phenotype of *T^hp^*^/+^ C57BL/6J-Y^AKR^ mice.

Here, we characterize the *T^hp^* deletion in more detail and estimate its minimal size and gene content. We also examine the *T^hp^*^/+^, B6.Y^AKR^ sex-reversal phenotype further, showing that sex reversal in this strain is more pronounced than in *Map3k4^tm1Flv^*^/+^, B6.Y^AKR^ mice. We show that reduced *Sry* expression at 11.5 dpc underlies XY gonadal defects in *T^hp^*^/+^ embryos. We then describe an *in vivo* gain-of-function genetic experiment using a mouse line carrying a functional *Map3k4* bacterial artificial chromosome (BAC) transgene. When introduced onto the *T^hp^*^/+^, B6.Y^AKR^ genetic background this transgene completely rescues the *Tas* sex-reversal phenotype by re-establishing a normal *Sry* expression profile. We conclude from these data that: (i) *Map3k4* haploinsufficiency is necessary for *T*-associated sex reversal, (ii) some interaction between reduced *Map3k4* dosage and another genetic deficiency in *T^hp^*^/+^ mice exacerbates the sex-reversal defect in *T^hp^*^/+^ embryos when compared with *Map3k4^tm1Flv^*^/+^ embryos and (iii) *Map3k4* is a major determinant of the timing and levels of *Sry* expression in developing mouse gonads.

## RESULTS

### Further molecular characterization of the *T^hp^* deletion

In order to get a more precise estimate of the gene content of the *T^hp^* deletion, we characterized its genomic location in greater detail. We used a strategy based on quantitative polymerase chain reaction (qPCR) to determine the copy number of genes on proximal chromosome 17 in the vicinity of *T*, *Map3k4* and *Igf2r* (all known to reside in the deletion) in *T^hp^*^/+^ C57BL/6J-Y^AKR^ mice and thereby determine the distal limits of the deletion (see Materials and Methods; Supplementary Material, Fig. S1 for more details). The position of known deleted loci allowed us to estimate a minimal size for the deletion of 5.56 Mb. This minimal region (based on *Ensembl* mouse genome database build GRCm38.p1) is predicted to contain 62 genes. A maximum size of the deletion, based on non-deleted loci, is 6.34 Mb, containing 84 genes. None of the genes within either the calculated maximal or minimal deleted regions, with the exception of *Map3k4*, has been implicated in testis determination. A more precise delineation of the deletion break points was prevented by regions that contain highly homologous sequences, immediately adjacent to the minimally deleted region (Supplementary Material, Fig. S1). Illegitimate recombination between these repeated regions may offer a mechanistic explanation of the original deletion event itself.

### Ovotestis and ovary development in XY *T^hp^*^/+^ and *Map3k4^tm1Flv^*^/+^ embryos at 14.5 dpc

**Figure 1.**
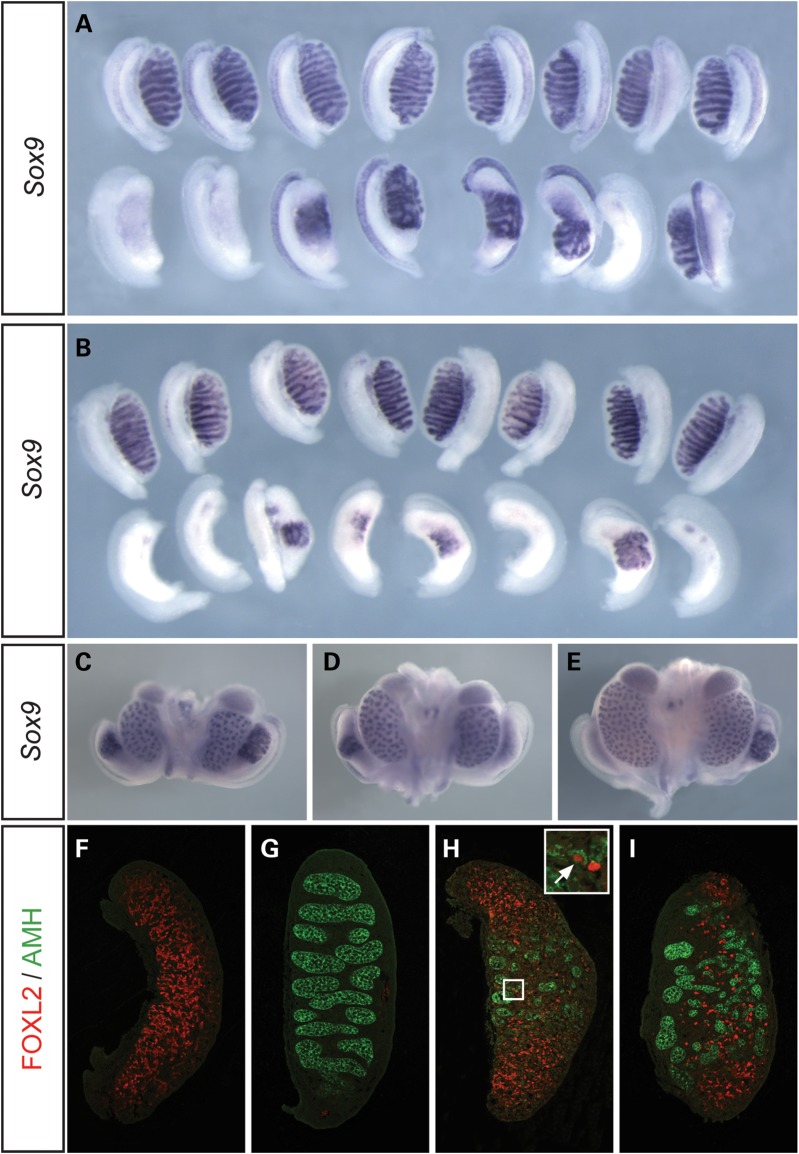
Abnormalities of testis development in XY *Map3k4^tm1Flv^*^/+^ and XY *T^hp^*^/+^ embryos at 14.5 dpc. (**A**) WMISH analysis of wild-type (+/+, upper row) and *Map3k4^tm1Flv^*^/+^ (lower row) embryonic gonads on B6.Y^AKR^ using a *Sox9* probe, showing XY ovary or ovotestis development in *Map3k4^tm1Flv^*^/+^ embryos at 14.5 dpc. (**B**) *Sox9* WMISH analysis of wild-type (upper row) and *T^hp^*^/+^ (lower row) embryonic gonads on B6.Y^AKR^, revealing ovary and ovotestis development in XY *T^hp^*^/+^ embryos with apparent enhanced severity. (**C–E**) *Sox9* WMISH of *Map3k4^tm1Flv^*^/+^ embryonic urogenital organs at 14.5 dpc revealing variable *Sox9* expression and gonad morphology even within individual embryos. Embryos may show ovotestes on both sides (C), ovotestis and ovary development on the right and left side, respectively, as in (D), or the converse (E). (**F–I**) Immunostaining of gonadal tissue sections for FOXL2 (red) and anti-müllerian hormone (AMH) (green) at 14.5 dpc. Images show control (+/+) XX gonad exhibiting only FOXL2 expression (F); control XY gonad exhibiting only AMH expression in testis cords (G); *Map3k4^tm1Flv^*^/+^ gonad with central AMH expression and polar FOXL2 expression (H). Note FOXL2-positive cells in interstitium of the central testicular region and an individual cell positive for both FOXL2 and AMH (inset, white arrow); (I) *T^hp^*^/+^ ovotestis tissue section with cellular distribution similar to (H) .

We began our phenotypic analysis of *T*-associated sex reversal in XY *T^hp^*^/+^ and *Map3k4^tm1Flv^*^/+^ mice by examining embryonic sexual development at 14.5 dpc, a stage at which it is easy to detect the presence of ovotestes (Fig. 1). These have previously been described as a common feature of XY *T^hp^*^/+^ embryos and are thought to be a cause of the hypoplastic testes observed in adult males (21). *T^hp^*^/+^ embryos examined were produced after five generations or more of crossing *T^hp^*^/+^ males to B6, allowing a more appropriate comparison with the embryonic gonadal phenotype of *Map3k4^tm1Flv^*^/+^ heterozygotes, which were congenic on B6.

At 14.5 dpc, analysis of gonadal *Sox9* expression by whole mount *in situ* hybridization (WMISH) revealed varying levels of transcript in both *Map3k4^tm1Flv^*^/+^ and *T^hp^*^/+^ embryos (Fig. 1A and B). *Map3k4^tm1Flv^*^/+^ gonads were frequently ovotestes and exhibited *Sox9* expression at high levels only in the centre of the gonad, in association with testis cords, as previously described (26). Dysgenetic testes, with varying numbers of irregular testis cords, were commonly observed. Occasionally, there was greatly reduced *Sox9* expression and the gonad had an overtly ovarian appearance (Fig. 1A). Analysis of XY *T^hp^*^/+^ embryos at the same stage revealed a higher frequency of gonads with an overt ovarian morphology and negligible *Sox9* expression, in addition to ovotestes (Fig. 1B). *Sox9*-positive regions of these ovotestes had less well-defined testis cord structures than XY *Map3k4^tm1Flv^*^/+^ equivalents. Interestingly, in addition to variation between individual embryos, variation in the degree of *Sox9* expression between gonads from individual XY *Map3k4^tm1Flv^*^/+^ embryos was also common, indicating a non-genetic contribution to the variability in the gonadal phenotypes observed (Fig. 1C–E). The apparent increased frequency and severity of gonadal sex reversal observed in *T^hp^*^/+^ embryos when compared with *Map3k4^tm1Flv^*^/+^ is consistent with our observations of the adult phenotype of these lines. *Map3k4^tm1Flv^*^/+^ and *Map3k4^byg^*^/+^ XY males commonly had reduced testis weights but were infrequently scored as phenotypic females (∼15%, data not shown). In contrast, XY *T^hp^*^/+^ mice exhibited a higher frequency of XY sex reversal (∼40%, data not shown). Morphological abnormalities of the reproductive tracts were common in these adult XY *T^hp^*^/+^ mice, including those scored as males (data not shown).

Previous studies of ovotestis development in other genetic models have revealed a fine-grain mixing of ovarian and testicular cell types beneath the coarser testicular and ovarian domains identified morphologically: FOXL2-positive cells have been identified in testicular interstitial regions of B6 XY*^POS^* ovotestes and SOX9- or anti-müllerian hormone (AMH)-positive cells detected in ovarian regions of XX *Wt1:Sox9* transgenic ovotestes (29,30). These studies revealed a mutually exclusive pattern of FOXL2 and SOX9 protein expression at the cellular level, reflecting the antagonistic relationship of these molecules and their respective pathways during sex determination. However, a third study reported co-expression of FOXL2 and AMH at the cellular level in ovotestes generated by haploinsufficiency for testis-determining genes in the B6.YAKR gonad (16). We examined *Map3k4^tm1Flv^*^/+^ and *T^hp^*^/+^ ovotestes at 14.5 dpc with antibodies to FOXL2 and AMH in order to examine the distribution of cells committed to the ovarian and testicular fates, respectively (Fig. 1F–I). AMH was detected exclusively in wild-type XY gonads at 14.5 dpc, and FOXL2 exclusively in XX wild-type gonads. In ovotestes from both strains the central, testicular regions exhibited strong AMH expression in testis cords, whilst the gonadal poles showed equally high levels of FOXL2. However, large numbers of FOXL2-positive cells were observed in the testicular region, although these were excluded from well-formed testis cords. AMH-positive cells were not detected in the ovarian poles. Very rarely, an individual cell appeared to express both FOXL2 and AMH (inset, Fig. 1H).

### *Sox9* and *Sry* expression is disrupted in *T^hp^*^/+^ and *Map3k4^tm1Flv^*^/+^ embryonic gonads

**Figure 2.**
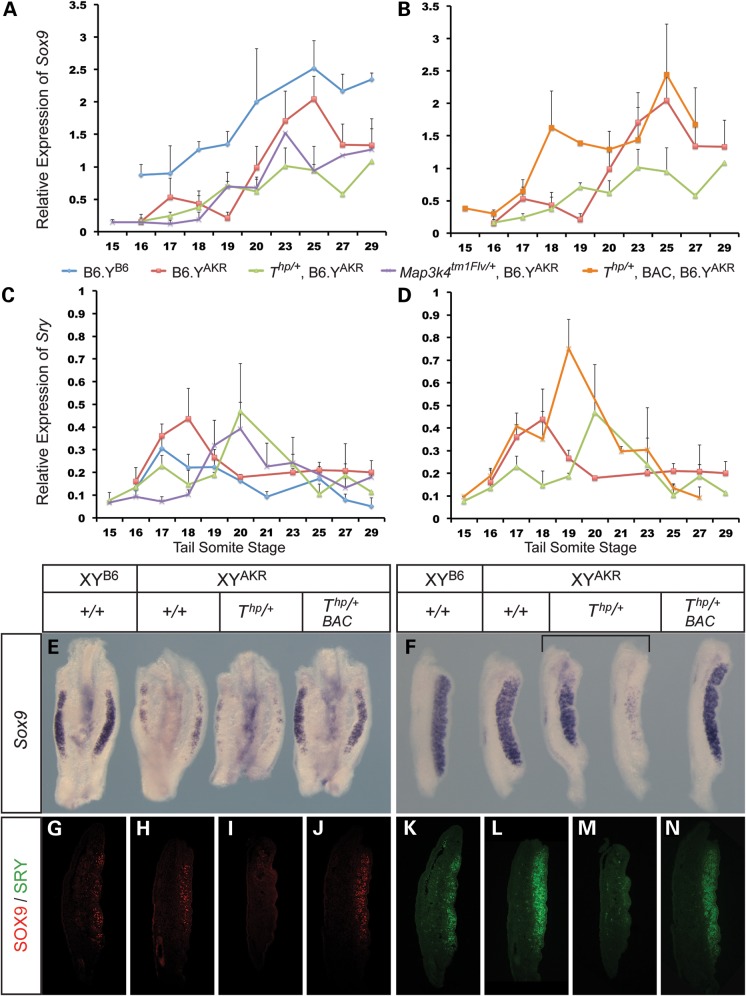
Expression profiling of *Sox9* and *Sry* expression in XY *Map3k4^tm1Flv^*^/+^, *T^hp^*^/+^ and transgenic *T^hp^*^/+^ embryonic gonads. (**A** and **B**) Relative expression levels of *Sox9* for different genotypes (indicated by colour key beneath plots) between 15 ts and 29 ts stages. (**C** and **D**) Relative expression levels of *Sry* in gonads from the same genotypic classes and stages as in A and B. Note the delay in peak *Sry* expression in *Map3k4^tm1Flv^*^/+^ and *T^hp^*^/+^ gonads (**C**) and the enhanced, early *Sry* peak in transgenic *T^hp^*^/+^ gonads (D). Error bars indicate standard error mean. (**E**) *Sox9* WMISH analysis at 20 ts in genotypes as indicated. (**F**) *Sox9* WMISH analysis at 27 ts (∼12.5 dpc) in genotypes as indicated. (**G–J**) Anti-SOX9 antibody immunostaining of control B6.Y^B6^ (G), control B6.Y^AKR^ (H), *T^hp^*^/+^ (I) and rescued (BAC transgenic) *T^hp^*^/+^ (J) gonadal tissue sections at 18 ts. (**K**–**N**) Anti-SRY antibody immunostaining of the same series of 18 ts gonadal sections as in G–J.

To determine the molecular basis of ovotestis and ovary development in these mutant lines we began by carefully quantifying expression of *Sox9* during the sex-determining period of gonadogenesis, ∼11.25–12.5 dpc, using qRT-PCR. For comparison, we also included samples from B6.Y^B6^ and B6.Y^AKR^ wild-type gonads at the same stages. This expression profiling revealed that *Sox9* transcript levels rise slowly in B6.Y^AKR^, reaching peak levels ∼25 tail somites (ts) (Fig. 2A). Equivalent levels of *Sox9* were observed earlier, at 19–20 ts, in B6.Y^B6^ gonads. This altered profile is consistent with delayed testis cord formation in B6.Y^AKR^ gonads, which is associated with transient ovotestis development at 14.5 dpc (24). In *Map3k4^tm1Flv^*^/+^, B6.Y^AKR^ gonads, *Sox9* levels also remained very low until 18 ts; levels began to rise from ∼19 ts but failed to reach those observed at 25 ts in B6.Y^AKR^ controls. In contrast to this, *Sox9* levels remained comparatively low in *T^hp^*^/+^ gonads at all stages (Fig. 2A). However, in contrast, strong expression of the ovarian somatic marker *Wnt4* was detectable at 18 ts in *T^hp^*^/+^ gonads (Supplementary Material, Fig. S2). Variation in expression levels between individual tissue samples from the same mutant strains was common and likely underlies the phenotypic variability observed at 14.5 dpc. Combining data from multiple samples masks this variation.

These data suggest a delay in execution of the sex-determining programme in B6.Y^AKR^ gonads in comparison to B6.Y^B6^, as evidenced by *Sox9* expression, that is exacerbated further in *Map3k4^tm1Flv^*^/+^ and even more so in *T^hp^*^/+^ embryonic gonads. WMISH analyses at 11.75 dpc (20 ts, Fig. 2E) and 12.5 dpc (27 ts, Fig. 2F) using a *Sox9* probe corroborated the qRT-PCR data, revealing a reduction or delay in *Sox9* expression in *T^hp^*^/+^ that, in some gonads, is associated with spatial restriction of expression or negligible detectable levels by 12.5 dpc (Fig. 2F).

Given the reported role for SRY in regulation of *Sox9* expression in the mouse (31), and the significance attributed to reduction of, and delay in, *Sry* expression in models of B6.Y^DOM^ sex reversal (32,33), we next examined the expression profile of *Sry* in both mutant strains and controls by qRT-PCR at multiple developmental stages. In wild-type B6.Y^AKR^ gonads, *Sry* levels rose to a peak at 18 ts before slowly declining (Fig. 2C). Interestingly, a similar profile was observed in B6.Y^B6^ gonads but the *Sry^B6^* allele peaked earlier (at 17 ts) and did not reach the levels of *Sry^AKR^*. This reduced expression of *Sry^B6^* compared with *Sry^AKR^* in the B6 genetic background has been previously reported (33,34). Moreover, *Sry^B6^* expression dropped to lower levels than observed for *Sry^AKR^*, which remained higher throughout the time-course. In mutant *Map3k4^tm1Flv^*^/+^ and *T^hp^*^/+^ gonads, *Sry* rose slowly and did not reach levels equivalent to peak expression in wild-type B6.Y^AKR^ gonads until ∼20 ts; levels at 18 ts in both mutant strains were significantly reduced in comparison to B6.Y^AKR^ wild-type controls (Fig. 2C). Profiles of *Sry^AKR^* expression in *T^hp^*^/+^ and *Map3k4^tm1Flv^*^/+^ gonads did not differ substantially, although levels rose slightly earlier in *Map3k4^tm1Flv^*^/+^ gonads, resulting in significantly higher levels in this strain at 19 ts. This may account for an apparently reduced severity of sex-reversal phenotype in the *Map3k4^tm1Flv^*^/+^ *Tas* model. As in the case for *Sox9*, considerable variation between individual samples was observed. Overall, these data indicate a delay in *Sry* expression reaching peak levels in *Map3k4^tm1Flv^*^/+^ and *T^hp^*^/+^ gonads on B6.Y^AKR^, likely accounting for defects in testis determination in these strains.

### Functional *Map3k4* BAC transgenes rescue defects in *T^hp^*^/+^ gonads

**Figure 3.**
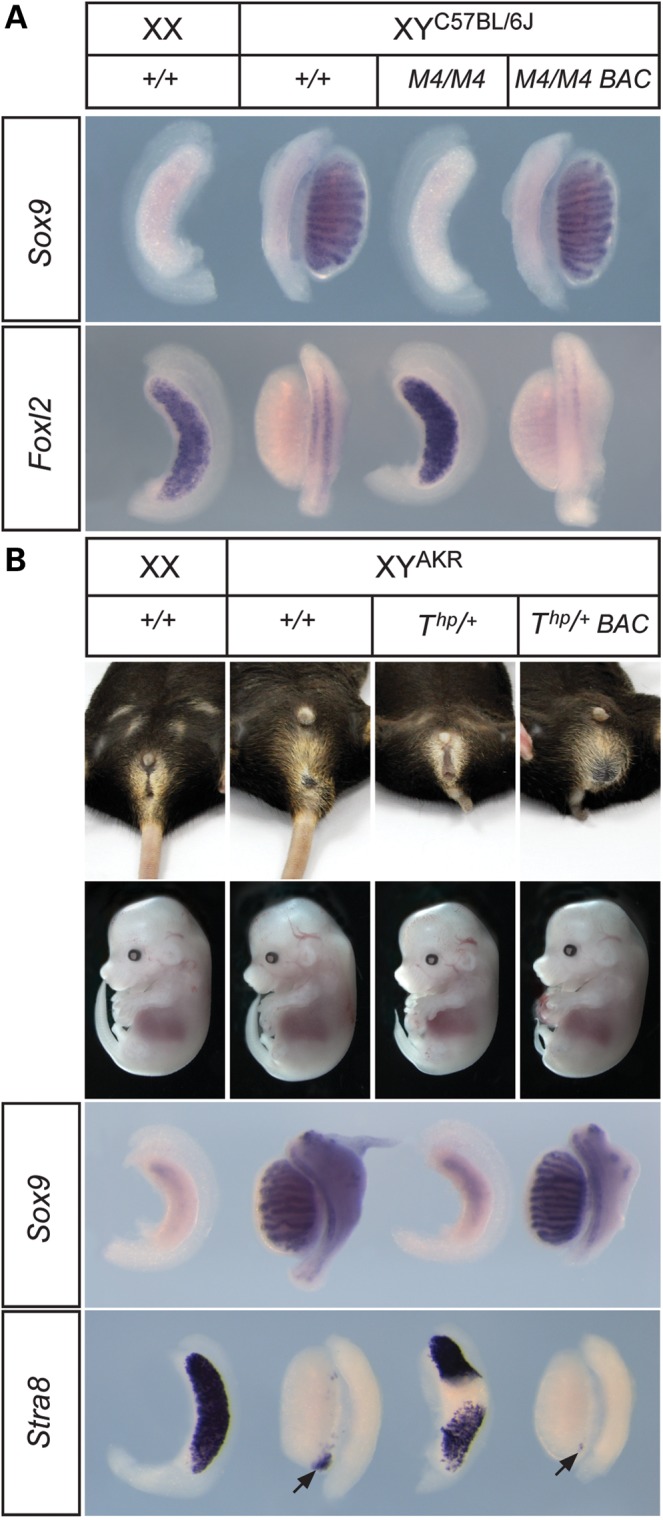
A functional *Map3k4* BAC transgene rescues abnormalities of XY gonad development in *T^hp^*^/+^ embryos. (**A**) WMISH analysis with *Sox9* (upper row) and *Foxl2* (lower row) of gonads at 14.5 dpc in control (+/+) and *Map3k4*-deficient (*M4/M4*) and transgenic *Map3k4*-deficient (*M4/M4*, BAC) embryos, revealing rescue of XY gonadal sex reversal in the latter class. (**B**) External genitalia of adult mice with genotypes indicated (upper row) and gross morphology of 14.5 dpc embryos with genotypes indicated (beneath). Note abnormal tails in *T^hp^*^/+^ animals. Lower panels show WMISH analyses with *Sox9* (upper) and *Stra8* (lower) of embryonic gonads at 14.5 dpc in genotypes indicated, revealing rescue of gonadal sex reversal in transgenic XY *T^hp^*^/+^ embryos. Arrows indicate clusters of *Stra8*-positive cells of varying sizes at the poles of the gonads.

*Map3k4* haploinsufficiency results in many of the gonadal abnormalities associated with *Tas*, although with an apparent reduced frequency and severity of sex reversal. In order to test whether *Map3k4* haploinsufficiency is absolutely required for *Tas*, or whether loss of other loci in the *T^hp^* deletion is also sufficient to generate a sex reversal phenotype, we performed a gain-of-function experiment utilizing a previously reported *Map3k4* BAC transgenic line, c06 (28) (Fig. 3), and lines harbouring an independent *Map3k4* BAC, k10 (data not shown). Each BAC contained the complete *Map3k4* transcriptional unit and varying amounts of 5′ and 3′ flanking DNA (for details see Materials and Methods). No other complete transcriptional units are predicted to reside within either BAC clone.

To test for functionality of the BACs in these lines, we generated embryos homozygous for the *Map3k4^tm1Flv^* targeted knockout that also carried a BAC transgene. XY embryos homozygous for *Map3k4^tm1Flv^* exhibit gonadal sex reversal, containing ovaries at 14.5 dpc (Fig. 3A). However, when the c06 and k10 (data not shown) BAC transgenes were present, *Map3k4^tm1Flv^* homozygous XY embryos (*n* = 3) developed testes with a normal morphology. WMISH with *Sox9* also revealed that Sertoli cell differentiation had occurred normally and examination of *Foxl2* expression demonstrated absence of ovarian tissue (Fig. 3A). Thus, we conclude that *Map3k4* expressed from either BAC transgene was sufficient to rescue the sex reversal phenotype in these homozygous mutant embryos. Prior qRT-PCR analysis of *Map3k4* expression levels in transgenic and wild-type gonads at 11.5 dpc had revealed that expression levels were ∼5-fold higher in transgenic (line c06) XY gonads at 11.5 dpc (28).

We then generated mice transgenic for the c06 BAC transgene that also carried the *T^hp^* deletion on B6.Y^AKR^. In contrast to the XY gonadal abnormalities usually associated with *T^hp^* on this genetic background, adult transgenic XY *T^hp^*^/+^ mice were all fertile males with overtly normal morphology of the reproductive organs (data not shown) and external genitalia (Fig. 3B). We then generated embryos in the control and rescued genotypic classes. Examination of XY embryonic gonads at 14.5 dpc also confirmed that the presence of the c06 BAC transgene suppressed ovotestis development in XY *T^hp^*^/+^ embryos and thus rescued the *Tas* phenotype. Marker gene expression in gonads from rescued embryos revealed prominent *Sox9* expression in testis cords at 14.5 dpc and absence of *Stra8*, a marker of meiotic germ cells that is ovary-specific at this stage (Fig. 3B). Equivalent data were generated using the k10 BAC transgene (data not shown). From these data we conclude that haploinsufficiency for *Map3k4* is key to development of the *Tas* phenotype.

To examine the molecular basis of rescue of the *Tas* phenotype during the sex-determining period we performed qRT-PCR profiling of *Sry* and *Sox9* in *T^hp^*^/+^ embryos harbouring the BAC transgene. Examination of *Sry* expression in transgenic *T^hp^*^/+^, B6.Y^AKR^ gonads revealed a positive shift in its profile in comparison to non-transgenic controls, with levels at 17 ts already comparable to the peak of expression observed in *T^hp^*^/+^ gonads at 20 ts (Fig. 2D). Moreover, levels continued to rise to a peak at 19 ts in transgenic *T^hp^*^/+^ gonads, greater even than those observed in control, wild-type B6.Y^AKR^ gonads, before dropping quickly to negligible levels by 27–29 ts. *Sox9* expression profiles in transgenic gonads also revealed early, robust expression, in contrast to *T^hp^*^/+^ gonads (Fig. 2B). Indeed, levels comparable to those found in non-transgenic, wild-type B6.Y^AKR^ controls at 23–25 ts were observed by 18 ts. This early surge of *Sox9* expression in rescued transgenic gonads, in addition to accounting for rescue of the *T^hp^*^/+^ phenotype, might account for an apparent acceleration in testis development in these samples when compared with wild-type B6.Y^AKR^ controls, as evidenced by the reduced expression of *Stra8* at the gonadal poles at 14.5 dpc (Fig. 3B). WMISH analysis also confirmed the rescue of robust *Sox9* expression in transgenic *T^hp^*^/+^ gonads at 19 ts (Fig. 2E) and 12.5 dpc (Fig. 2F). Moreover, immunostaining of sectioned gonads with an anti-SOX9 antibody revealed increased numbers of SOX9-positive cells in transgenic XY *T^hp^*^/+^ gonads at ∼18 ts in comparison to non-transgenic gonads from *T^hp^*^/+^ littermates (Fig. 2G–J). Finally, detection of SRY protein at the same stage also revealed large numbers of SRY-positive cells in rescued gonads when compared with *T^hp^*^/+^ equivalents (Fig. 2K–N). Thus, we conclude that transgenic expression of *Map3k4* rescues the *Tas* phenotype by re-establishing early, robust *Sry* and *Sox9* expression profiles that are disrupted in *T^hp^*^/+^ embryos.

## DISCUSSION

*T*-associated sex reversal (*Tas*) in the mouse was first described before the identification of *Sry* as the mammalian testis-determining gene (21). Subsequent studies on one particular *Tas* model phenotype, B6 *T^Orl^*^/+^ XY^AKR^, suggested severely reduced *Sry* expression as a possible cause of sex reversal (24). This, along with the development of normal testes in B6 *T^Orl^*^/+^ XY^AKR^ mice when a *Mus musculus*-derived *Sry* transgene is present, indicates that the *Sry^AKR^* allele is deficient on the B6 *T^Orl^*^/+^ genetic background due to abnormalities in the regulation of its expression. By inference, given the overlapping nature of the *T^Orl^* and *T^hp^* deletions, a similar mechanistic explanation was thought to account for the *T^hp^*^/+^ version of *Tas*.

Here, we confirm that *Sry* expression is disrupted in B6 *T^hp^*^/+^ XY^AKR^ embryos. Moreover, we demonstrate that MAP3K4 function is central to a mechanistic account of *Tas* by showing that *Map3k4* haploinsufficiency on B6.Y^AKR^ also results in disrupted *Sry* expression in a manner similar to that observed in *T^hp^*^/+^ gonads and that expression of *Map3k4* from a BAC transgene is sufficient to rescue *Tas* in the mouse. XY transgenic *T^hp^*^/+^ gonads show no signs of ovotestis or ovary development. This rescue is the result of early and robust *Sry* expression in transgenic gonads, in contrast to deletion carriers that up-regulate *Sry* expression later, with predicted detrimental consequences for the activation of *Sox9* expression and testis determination. Whilst, we cannot formally exclude a direct, positive effect of the *Map3k4* BAC transgene on testis-determining genes downstream of *Sry*, such as *Sox9*, the normal sexual development and fertility of XX transgenic females does not support such a model. Our previous studies on testis determination suggest that MAP3K4 signalling in gonadal somatic cells activates p38 MAPK and results in phosphorylation of GATA4, a transcription factor known to regulate expression of *Sry* (28). Thus, transgenic expression of *Map3k4* is presumed to rescue defects in this signalling pathway in XY *T^hp^*^/+^ gonads. We cannot formally exclude, however, other positive impacts of MAP3K4 on *Sry* expression or function.

The rescue of complete and partial XY gonadal sex reversal by the *Map3k4* transgene establishes *Map3k4* as the key gene in the *T^hp^* deletion in the aetiology of *T*-associated sex reversal (*Tas*). How, therefore, do we account for the apparent increased frequency and severity of XY gonadal sex reversal in *T^hp^*^/+^ carriers when compared with that observed in embryos heterozygous for a *Map3k4* null allele alone? In both cases, the copy number of *Map3k4* is the same. Three possible explanations are: (i) haploinsufficiency for another locus (or loci) in the *T^hp^* deletion, exacerbating the disruption to XY gonad development; (ii) disruption to a testis-determining gene outside, but close to, the *T^hp^* deletion in the form of a position effect or (iii) the presence of the deletion itself contributing to disruption to testis determination. All of these potential explanations rely on a form of genetic interaction that is asymmetrically dependent on *Map3k4* haploinsufficiency, since the proposed genetic deficits do not disrupt testis determination in the presence of the transgene. We cannot exclude any of these possibilities. With respect to the first possibility, our novel data concerning the extent of the *T^hp^* deletion indicate that it contains no other genes implicated in testis determination. However, further investigation may reveal a novel testis-determining function encoded by the deleted region. In the case of the second possibility, we have recently reported the role of p38α and p38β MAPK in mouse testis determination (28). *p38α* (*Mapk14*) and *p38δ* (*Mapk13*) map to proximal mouse chromosome 17, but we have shown that neither resides within the *T^hp^* deletion region and their expression levels are not significantly disrupted in XY *T^hp^*^/+^ gonads at 11.5 dpc (Supplementary Material, Fig. S3). With respect to the third explanation, the mere presence of hemizygous deletions in the mouse, quite apart from their actual gene content, has previously been invoked as an explanation of sex-reversal phenomena such as *Tas,* due to possible effects on embryonic growth (25). Here, we show that any contribution that the *T^hp^* deletion itself makes to *Tas*, independently of specific gene dosage alterations, is conditional on the haploinsufficiency of *Map3k4*.

The re-establishment of early, robust *Sry* expression at 17 ts in transgenic *T^hp^*^/+^ B6.Y^AKR^ gonads, and the increased levels attained when compared with wild-type XY^AKR^ controls, in addition to explaining the phenotypic rescue itself, might account for a small, but detectable, acceleration of testis development in these embryos when compared with B6.Y^AKR^ wild-type controls (compare, e.g. testis cord morphology and *Stra8* expression pattern in gonads shown in Fig. 3B). Transgenic gonads have an appearance and *Stra8* expression profile more reminiscent of B6 gonads at the same stage, rather than B6 XY^AKR^. This phenomenon suggests that MAP3K4 activity may be a limiting factor in the degree of up-regulation of *Sry* expression on the B6.Y^AKR^ background. This observed transgenic rescue is also not apparently hampered by SRY protein isoform differences between the *Sry^B6^* and *Sry^AKR^* alleles, which, along with altered *Sry* expression, have been implicated in the delay in B6 XY^AKR^ testis determination (32). While the disruption to *Sry* expression in *T^hp^*^/+^ and *Map3k4^tm1Flv^*^/+^ gonads is relatively subtle, it is well established that only minor alterations to the timing or levels of *Sry* expression can radically alter phenotypic consequences: ovary, ovotestes or dysgenetic testes can all arise depending on subtle changes to *Sry* expression control (17,33,35–37).

Phenotypic variation, even between individuals that are essentially isogenic, is a feature of the *Map3k4^tm1Flv^*^/+^ B6.Y^AKR^ gonadal phenotype. Crucially, asymmetric gonad morphology within individual embryos is observed, ruling out a residual genetic contribution to this variability, at least in these individuals. This variability is likely related to the reduction of MAP3K4 functionality to near-threshold levels, and the consequence of this for levels and timing of *Sry* expression during gonadogenesis. Developmental stochasticity is a research area receiving increased attention (38). A number of features of stochastic systems may be relevant in this context. Firstly, strong fluctuations can be a consequence of low numbers in any system. It is known that SRY-positive cells arise in the centre of the developing gonad at ∼10.75 dpc in low numbers (39). These first, potentially stochastic, events in gonadal SRY expression are likely to result in subsequent recruitment of further cells to the testis-determining (pre-Sertoli cell) pathway. In other systems, gene regulatory networks act to buffer stochastic variability in gene expression, and loss of particular genes can produce pronounced phenotypic variation, including variable penetrance, that reveals underlying stochasticity of gene expression (40,41). Loss of a single copy of *Map3k4*, on an already sensitized genetic background, appears to exacerbate inherent stochasticity in the testis-determining mechanism, although the precise events governed by this stochasticity are unknown. Phenotypic plasticity and stochastic noise have also been linked to epigenetic mechanisms (42). The significance of epigenetic regulation of the Y chromosome, in particular the *Sry* locus, for testis determination has recently been underlined by a report of XY gonadal sex reversal in mice lacking the histone demethylase, JMJD1A (43). Finally, these observations in mice suggest that stochasticity, in addition to variation in genetic background, may underlie some of the phenotypic variability between individuals with 46,XY pure or CGD caused by mutation of autosomal or X-linked human genes functioning in early steps of testis determination (27).

## MATERIALS AND METHODS

### Mouse mutants utilized and genotyping

All animal experimentation was approved by the Animal Welfare and Ethical Review Body at MRC, Harwell, and mice used in this study were bred with licensed approval from the UK Home Office (PPL 30/2877). Mice harbouring a targeted null mutation of *Map3k4* (*Map3k4^tm1Flv^*) have been previously described (44,45). *Map3k4^tm1Flv^*^/+^ mice were maintained on the C57BL/6J background and genotyped as previously described (26). *T^hp^*^/+^ mice were also maintained on C57BL/6J, and carriers identified by the distinctive shortened or kinked tail. This scoring method was verified by the use of a copy-number assay. Real-time PCR using *Taqman* chemistry permitted a copy number call of the brachyury gene (*T*), which is within the *T^hp^* deletion. Amplification of *T* was normalized relative to the endogenous control *Dot1L*. The ABI 7500 fast SDS software was used to determine the relative copy number of *T*. This approach was also employed to test other genes for residency within the deleted region. Primer sequences for all loci tested are shown in Supplementary Material, Table S1.

In experiments using the BAC transgene, the presence of the BAC was confirmed by amplification from the chloramphenicol resistance gene in the backbone of the BACe3.6 vector using the following primer pair: 5′-GCGTGTTACGGTGAAAACCT-3′ and 5′-GGGCACCAATAACTGCCTTA-3′.

Adult mice and embryos were sexed by a PCR assay that simultaneously amplifies the *Ube1y1* and *Ube1x* genes, using the following primer pair: 5′-TGGATGGTGTGGCCAATG-3′ and 5′-CACCTGCACGTTGCCCTT-3′ (46). The presence of the Y^AKR^ chromosome was confirmed by a PCR assay as previously described (26).

### BAC transgenesis

Identification of BAC clones: NOD/MrkTac BAC clones bQ279c06 (c06) and bQ285k10 (k10) were sourced from the Centre for Applied Genomics, Toronto, Canada. c06 comprised 178.7 kb of DNA including 78.7 kb of DNA 5′ and 8.8 kb 3′ of the *Map3k4* transcriptional unit. Clone k10 was 139.7 kb in length, including 30.3 kb 5′ and 9.4 kb 3′ of *Map3k4*. No other complete transcriptional units are predicted to reside in these BACs. The NOD/MrkTac mouse BAC library end sequences were mapped to the NCBIM37 mouse assembly using SSAHA alignment of the reads. No amino acid differences exist between the NOD/MrkTac- and C57BL/6J-encoded versions of MAP3K4. BAC DNA was prepared and injected into C57BL/6J 1-cell embryos to produce transgenic founders as described in (47).

### Generation of embryos and expression analyses

Noon on the day of the copulatory plug was counted as 0.5 dpc. Embryos were staged accurately based on the number of tail somites or limb and gonad morphology. WMISH analysis of embryonic tissues was performed as previously described (48,49). Probes for *Sox9* (50), *Sry* (51), *Wnt4* and *Stra8* (26,52) have been previously described. A 540 bp *Foxl2* probe was generated using primers 5′-AGAACGTGTCTGGTCGCTCT-3′ and 5′-GATCCGGGGAAATTTGTTTT-3′.

### Quantitative RT-PCR

Total RNA was extracted using RNeasy plus micro kit (Qiagen) from gonads separated from the mesonephros. RT was carried out with 150 ng of total RNA using the high-capacity cDNA RT kit (Applied Biosystem). qRT–PCR was performed with Fast SYBR Green Master Mix (Life technologies) on a 7500 Fast Real-Time PCR system (Applied Biosystem). RNA expression levels were normalized to those of *Hrpt1* (endogenous control) using the ΔΔCt method. At least three samples for each genotype were analysed. Primer sequences are shown in Supplementary Material, Table S1.

### Immunohistochemistry

Antibodies to the following proteins were utilized in this study: SOX9 (Millipore, #AB5535); AMH (Santa Cruz, #sc28912); FOXL2 (a kind gift from Dagmar Wilhelm and Peter Koopman). A polyclonal anti-SRY antibody was generated by first expressing a poly-histidine-tagged recombinant protein containing amino acids 82–395 of mouse SRY (UniProt accession no. Q05738) in *Escherichia coli* BL21 (DE3). Protein was purified using TALON resin (Clontech), mixed sufficiently with Freund's complete adjuvant to give a suspension and then injected intradermally into guinea pig (female, Hartley). Anti-SRY antibody was affinity-purified from serum using antigen. Immunostaining was performed on sectioned, paraffin wax-embedded tissue using the above primary antibodies (1:100) and Alexa Fluor 594 (FOXL2, SOX9) or 488 (AMH, SRY) conjugated secondary antibodies (1:200). Images were captured using a Zeiss 710 multiphoton microscope.

## SUPPLEMENTARY MATERIAL

Supplementary Material is available at *HMG* online.

## FUNDING

This work was supported by the United Kingdom Medical Research Council by Core funding to A.G. at the Mammalian Genetics Unit, Harwell (MC_A390_5RX50). Funding to pay the Open Access publication charges for this article was provided by the Medical Research Council.

## Supplementary Material

Supplementary Data
