# Supplementary material for: Transgenic expression of Map3k4 rescues T-associated sex reversal (Tas) in mice
Source: Hum Mol Genet. 2014 Jan 22;23(11):3035–44. doi: 10.1093/hmg/ddu020 (PMC4014197; doi:10.1093/hmg/ddu020)
Supplement: Supplementary Data [file supp_23_11_3035__index.html]

Transgenic Expression of Map3k4 Rescues T-associated Sex Reversal (Tas) in Mice — Transgenic expression of Map3k4 rescues T-associated sex reversal (Tas) in mice — Transgenic expression of Map3k4 rescues T-associated sex reversal (Tas) in mice — Supplementary Data 

# Transgenic expression of *Map3k4* rescues *T*-associated sex reversal (*Tas*) in mice

## Supplementary Data

Supplementary Data

**Files in this Data Supplement:**

- Supplementary Data - Doc file
- Supplementary Figure 1 - tif file
- Supplementary Figure 2 - tif file
- Supplementary Figure 3 - tif file
