# Supplementary material for: Transgenic expression of Map3k4 rescues T-associated sex reversal (Tas) in mice
Source: Hum Mol Genet. 2014 Jan 22;23(11):3035–44. doi: 10.1093/hmg/ddu020 (PMC4014197; doi:10.1093/hmg/ddu020)
Supplement: Supplementary Data [file supp_ddu020_ddu020supp.doc]

**Figure S1. Characterisation of the *T^hp^* deletion interval using quantitative PCR (qPCR) analysis of genes on proximal mouse chromosome 17.** A) Relative copy number of selected loci/amplicons defined by qPCR. qPCR data were normalised such that +/+ control samples contain two copies in each case. One copy per genome indicates haploinsufficiency and so inclusion in the *T^hp^* deletion. Error bars indicate SEM. B) Schematic of mouse chromosome 17 showing approximate position of the *T^hp^* deletion in A1. C) Approximate physical map of chromosome 17 from 7 Mb to 14 Mb showing the location of loci that were tested by qPCR in +/+ and *T^hp^*/+ animals**.** D) Based on qPCR data the regions of the chromosome not deleted in *T^hp^* are shown by thick black lines; regions deleted (assuming contiguity of deleted regions detected) are shown by a dotted line; regions for which data could not be definitively generated due to repeated sequences (indeterminate regions, IRs) are shown as dashed lines. Based on the *Ensembl* mouse genome database (GRCm38.p1) a minimum deletion size of 5.56 Mb is predicted – from position 7449353 (7.45 Mb) to position 13008354 (*Sod2*). The maximum calculated deletion size is 6.34 Mb – from position 7335656 (*Tcp10a*) to 13675715 (*Tcte2*). E) Schematic showing expanded view of IRs indicating position of tested loci and several blocks of highly homologous sequences: filled black arrows (between 7.30 to 7.42, 13.04 to 13.18 and 13.33 to 13.45 Mb) and filled grey arrows (13.18 to 13.22 and 13.45 to 13.52 Mb). Three regions corresponding to gaps in the contig assembly are also shown (open rectangles), between 7.47 to 7.52, 13.25 to 13.31 and 13.59 to 13.66 Mb. Line thickness corresponds to (D) above.

**Figure S2. Quantitative RT-PCR (qRT-PCR) reveals elevated expression of *Wnt4* in XY *T^hp^*/+ gonads.** Relative expression levels of *Wnt4* in embryonic gonads in the genotypic classes (colour key) and tail-somite (ts) stages indicated. Error bars show SEM.

**Figure S3. Quantitative RT-PCR (qRT-PCR) analysis of *Mapk13* and *Mapk14* expression in XY *T^hp^*/+ gonads at 11.5 dpc.** Relative expression levels of *Mapk13* and *Mapk14* in embryonic gonads in B6.Y^AKR^ wild-type (black) and *T^hp^*/+ (grey) gonads at 11.5 dpc. Error bars show SEM.

**Supplementary Table 1** Sequences of all primers used in this study to perform quantitative polymerase chain reaction (qPCR), either from reverse-transcribed RNA (*Hprt1, Sry, Sox9, Wnt4*) or genomic DNA.

|  | **Primers for qPCR** | | |
| --- | --- | --- | --- |
|  | **Locus** | **Forward primer** | **Reverse primer** |
|  | *Hrpt1* | CATTATGCCGAGGATTTGGAA | CACACAGAGGGCCACAATGT |
|  | *Sox9* | AAGAAAGACCACCCCGATTACA | CAGCGCCTTGAAGATAGCATT |
|  | *Sry* | TTATGGTGTGGTCCCGTGGT | GGCCTTTTTTCGGCTTCTGT |
|  | *Wnt4* | CTGGAGAAGTGTGGCTGTGA | GGACGTCCACAAAGGACTGT |
|  | *Rps6ka2* | AAAGGGATCCTTAGCCCAGA | AGGTTAGATGCCGAGAGCAA |
|  | *Tcp10a* | CCTTGATGACAAGGTCTGTAAGAAA | CCTGCTTACCACATTTATACCCTAT |
|  | 7.45 Mb | ACAGTGCGAGTGGGAAGAAC | TCAGGGATACACACGACCAA |
|  | *Fndc1* | TTGGACACATTGGTTTTGGA | CCCTAGCGCAGGTTAGTCAC |
|  | *Fgf1op* | TGGATGGAGAAACCACGATT | GCCCAAGTTTATGGTCGGTA |
|  | *Ccr6* | CCTGCAGTTCGAAGTCATCA | GGCAGCTGCAGTTTCTCTCT |
|  | *Mpc1* | AGCCTGAAGGCAGTTGTGTT | AGCCTGAAGGCAGTTGTGTT |
|  | *T2* | GGGAGCCAGTCTTGTCTTTG | GGAGCTACTGACTGCCGTTC |
|  | *T2'* | GGGTTGGTGTGTCCTGAAGT | GGCTTAAGTGCACCCACATT |
|  | *T* | CTGGGAGCTCAGTTCTTTCG | CCCCTTCATACATCGGAGAA |
|  | *Pde10a* | TGAGCAAGGAGAGGAGGTGT | TGTCCTGATTCCAGGAAAGG |
|  | *Gm17728* | GTGGTTTGCTGTGTGTGTCC | ACACTCTGTTGGGCCTGTTC |
|  | *Qk* | AGATGGCTCAGGAGGTGAGA | ATTGGAAGTGAGGCTGCAAG |
|  | *Pacrg* | AGAGCACACCCTCATCCATC | GAAGCATCTGGGTCTCAAGC |
|  | *Park2* | GCGTGGGAACTACTTGCTTC | TCTACAGCACAGGGTGGTGA |
|  | *Park2’* | TGACCCTGGACCATTCATTT | TTTCGAGCACAGTCACCTTG |
|  | *Map3k4* | GCTGATGCAGATAGCACGAA | GCTGAGGAAGGTGAGTCTGC |
|  | *Tcp1* | ATGGCATCCTAGAGCTTGGT | TCAGCCAGCTCACACAGAAC |
|  | *Sod2* | CCGAGGAGAAGTACCACGAG | CTGTGTTTTCCGGGATAGGA |
|  | *Tcte2* | GGCTTGTGACATGGGTCTTT | TCTCTGCTCTTCCCCTTCAA |
|  | *Mapk14* | TGCCCTTGACTAGGCATCTT | CGAGGTAGACTTCCCTGTGC |
|  | *Mapk13* | CCCTTTCCTCAGACAGCTTG | ACCACATAGCCCGTCATCTC |
|  |  |  |  |
