# Supplementary figures and images for: Transgenic expression of Map3k4 rescues T-associated sex reversal (Tas) in mice
Source: Hum Mol Genet. 2014 Jan 22;23(11):3035–44. doi: 10.1093/hmg/ddu020 (PMC4014197; doi:10.1093/hmg/ddu020)

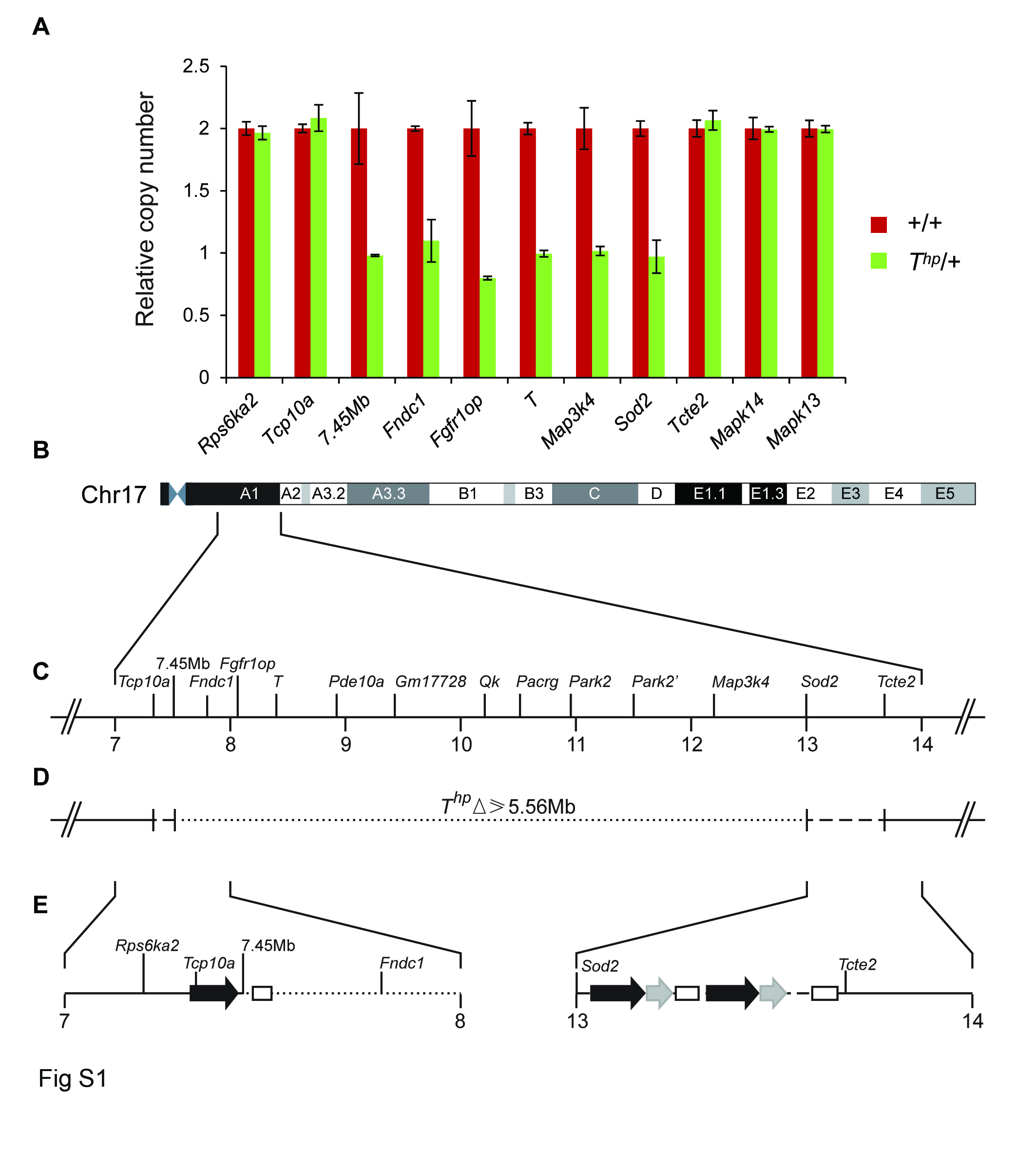

Supplement: Supplementary Data [file supp_ddu020_ddu020supp_fig1.tif]

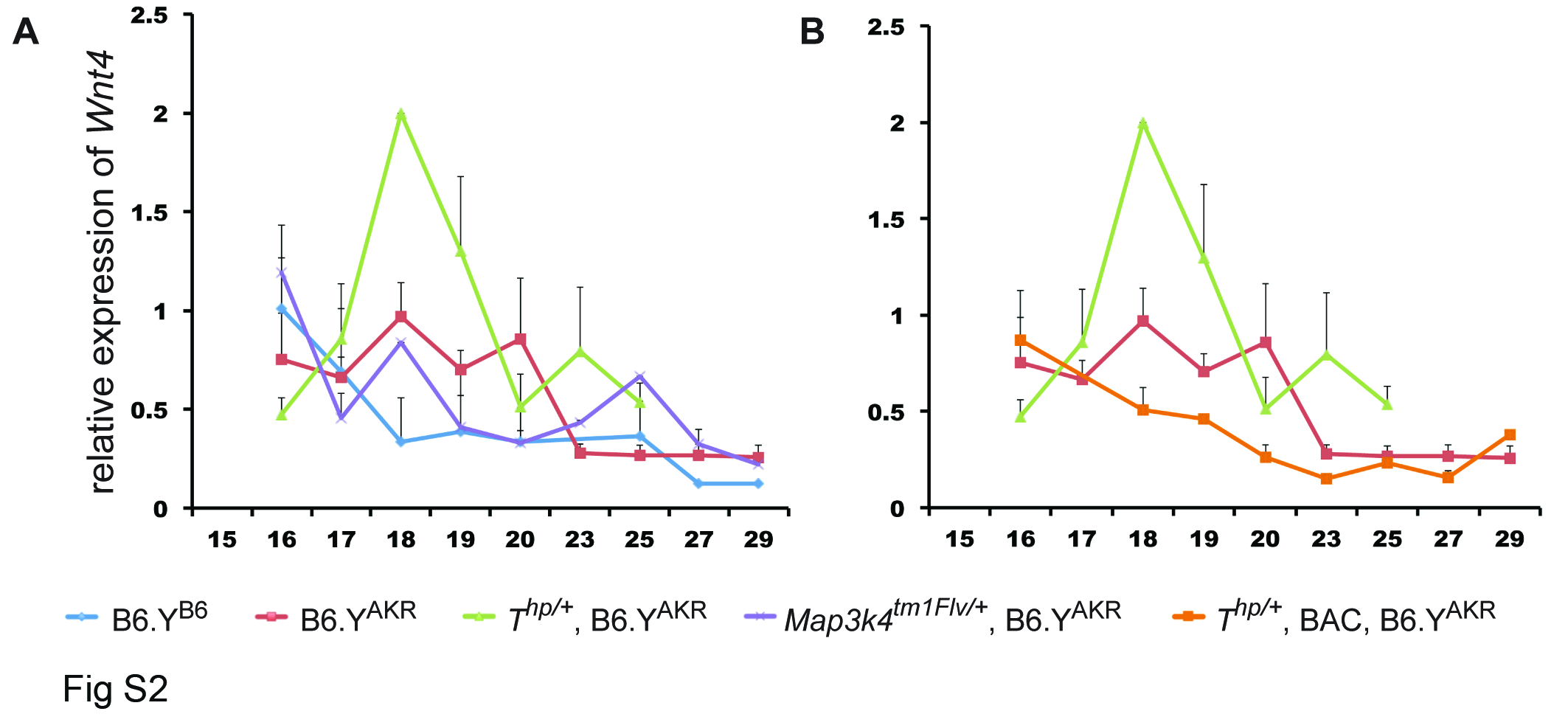

Supplement: Supplementary Data [file supp_ddu020_ddu020supp_fig2.tif]

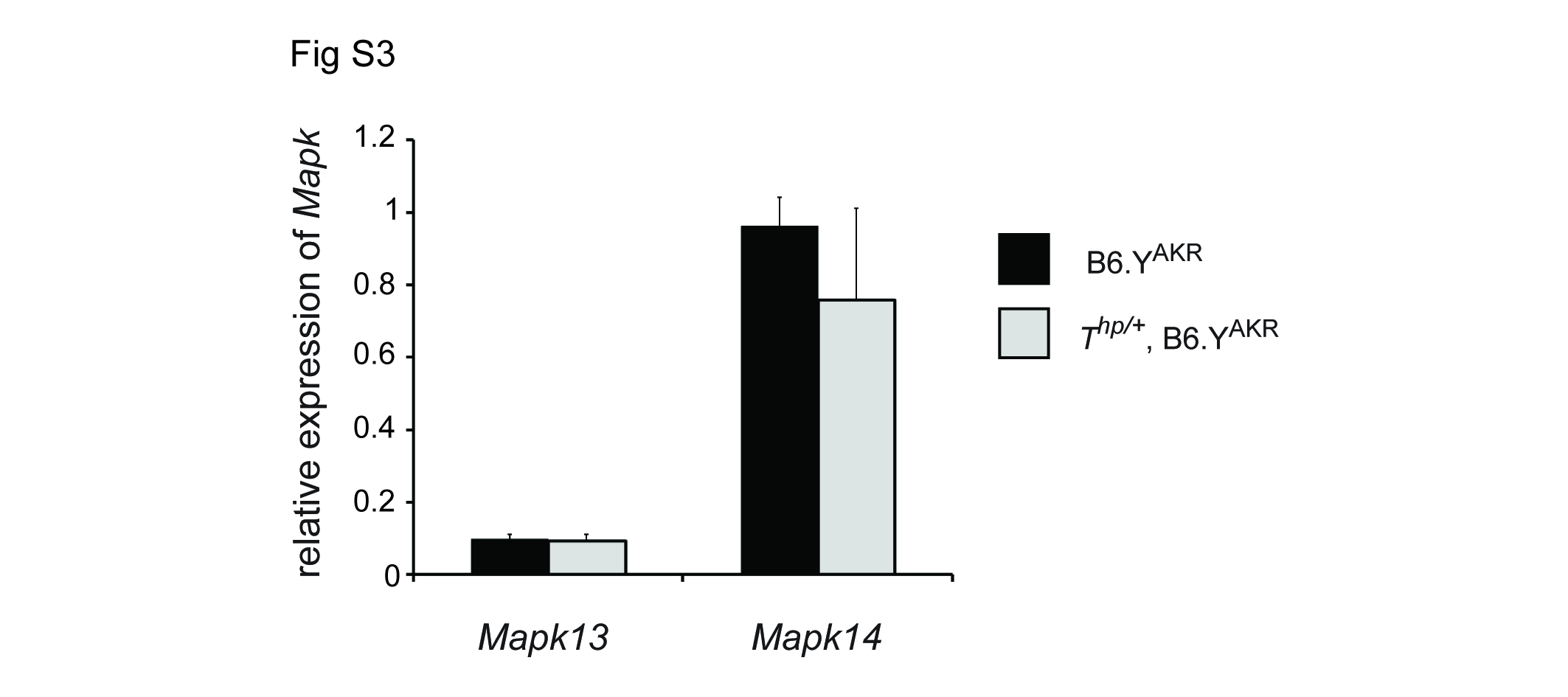

Supplement: Supplementary Data [file supp_ddu020_ddu020supp_fig3.tif]
